# Regeneration of neural crest derivatives in the *Xenopus *tadpole tail

**DOI:** 10.1186/1471-213X-7-56

**Published:** 2007-05-24

**Authors:** Gufa Lin, Ying Chen, Jonathan MW Slack

**Affiliations:** 1 Centre for Regenerative Medicine, Department of Biology and Biochemistry, University of Bath, Bath BA2 7AY, UK; 2 Stem Cell Institute, University of Minnesota, MTRF, 2001 6th Street SE, Minneapolis, MN 55455, USA

## Abstract

**Background:**

After amputation of the *Xenopus *tadpole tail, a functionally competent new tail is regenerated. It contains spinal cord, notochord and muscle, each of which has previously been shown to derive from the corresponding tissue in the stump. The regeneration of the neural crest derivatives has not previously been examined and is described in this paper.

**Results:**

Labelling of the spinal cord by electroporation, or by orthotopic grafting of transgenic tissue expressing GFP, shows that no cells emigrate from the spinal cord in the course of regeneration.

There is very limited regeneration of the spinal ganglia, but new neurons as well as fibre tracts do appear in the regenerated spinal cord and the regenerated tail also contains abundant peripheral innervation.

The regenerated tail contains a normal density of melanophores. Cell labelling experiments show that melanophores do not arise from the spinal cord during regeneration, nor from the mesenchymal tissues of the skin, but they do arise by activation and proliferation of pre-existing melanophore precursors. If tails are prepared lacking melanophores, then the regenerates also lack them.

**Conclusion:**

On regeneration there is no induction of a new neural crest similar to that seen in embryonic development. However there is some regeneration of neural crest derivatives. Abundant melanophores are regenerated from unpigmented precursors, and, although spinal ganglia are not regenerated, sufficient sensory systems are produced to enable essential functions to continue.

## Background

Most adult frogs do not regenerate missing parts, but their tadpoles often do [1,2]. In particular, the tadpole of *Xenopus laevis *will regenerate its tail after transection [3]. The new tail grows with a typical tapered form, and like the original tail contains a spinal cord, notochord and muscle. Because of the wealth of knowledge about *Xenopus *development, and the ease of micromanipulation of both embryonic and larval stages, this system is becoming an important model for the study of regeneration behaviour in animals [4-7]. Our own previous work has shown some differences from the regeneration of the urodele tail [8,9], in particular in the *Xenopus *tadpole there is no detectable de-differentiation and no metaplasia of spinal cord, notochord or muscle during regeneration. The spinal cord and notochord both regenerate from the corresponding tissue in the stump, and the satellite cells in the stump are the source of the new muscle mass in the regenerating tail [3,10]. In the present work we have examined the regeneration behaviour of another important group of tissues: the derivatives of the neural crest.

Originating from the border of the neural plate during early neurogenesis, the neural crest is a special embryonic cell population endowed with migratory capacity and the ability to form several differentiated cell types [11-14]. In embryonic development, the neural crest arises as a result of inductive interactions between the epidermis and the neural plate. Secreted factors such as Wnt proteins, bone morphogenetic proteins (BMPs) and fibroblast growth factors (FGFs) are all involved in this process [15-19]. But during regeneration there is no contact between the epidermis and the neuroepithelium of the spinal cord. Instead the end of the spinal cord closes to form a swollen vesicle known as the neural ampulla and the epidermis heals across the apex of the regeneration bud [2]. Given the absence of the anatomical condition for induction of neural crest we have asked two simple questions about this system:

a) Which neural crest-derived structures are replaced during regeneration?

b) What is their cellular origin?

The neural crest forms a variety of cell types [11,14,20,21]. These include the skeletal tissues of the head, part of the outflow tract of the heart, the enteric ganglia, the adenal medulla and several other tissue types. In the tail the main derivatives of the neural crest are the pigment cells and the spinal (dorsal root) ganglia containing sensory neurons with associated glia. The most prominent pigment cells in the *Xenopus *tail are the melanin-containing melanophores. Amphibian melanophores are very similar to those of fish whose development and regeneration has been studied in some detail [22-25]. It is conventional to refer to "melanophores" in lower vertebrates and "melanocytes" in amniotes but there is little if any difference between these cell types. Numerous melanophores are found in the *Xenopus *tadpole tail and we show here that they are regenerated and are very numerous in the new tail.

The spinal ganglia of all vertebrates are the neural crest-derived condensations of sensory neurons on the dorsal root of each spinal nerve [26]. The spinal nerves of *Xenopus *follow the primitive pattern, with one pair per myotome [27]. The spinal ganglia of the trunk (i.e. the region of the body retained after metamorphosis) are larger than those of the tail, and those of the tail may not be distinct from each other [28,29]. Here we show that the spinal ganglia are not re-formed during tail regeneration. The regenerate does however possess some sensory innervation because there are dorsally located neurons in the regenerated spinal cord, and there is a network of innervation detectable by antibody staining and by retrograde labelling of the nerve fibres reaching the skin. So the answer to question a) is that regeneration of neural crest derivatives in the *Xenopus *tail is incomplete, with good regeneration of melanophores but poor regeneration of spinal ganglia.

Question b) then becomes one about the origin of the melanophores of the regenerated tail. We have examined the cell lineage of the melanophores using various types of grafting and labelling experiment. Using two methods for labelling we show that cells are not exported from the spinal cord during regeneration. We show that melanophores are labelled by skin grafts but are not labelled by equivalent embryonic grafts lacking neural crest. Study of neural crest-ablated tadpoles shows that the melanophores regenerate from pre-existing melanophore precursors near the amputation surface, by proliferation and migration. This mechanism is similar to that described for the zebrafish [30,31].

Our overall conclusion is that the regeneration of neural crest derivatives in *Xenopus *is incomplete. Melanophores regenerate from unpigmented melanophore precursors. Sensory neurons regenerate within the spinal cord but there is little or no regeneration of spinal ganglia. The results show that the regenerated tail, although it has the basic shape and functional capabilities of a tail, is not an exact replica of the original tail.

## Results

### Labelled cells do not leave the spinal cord

**Figure 1:**
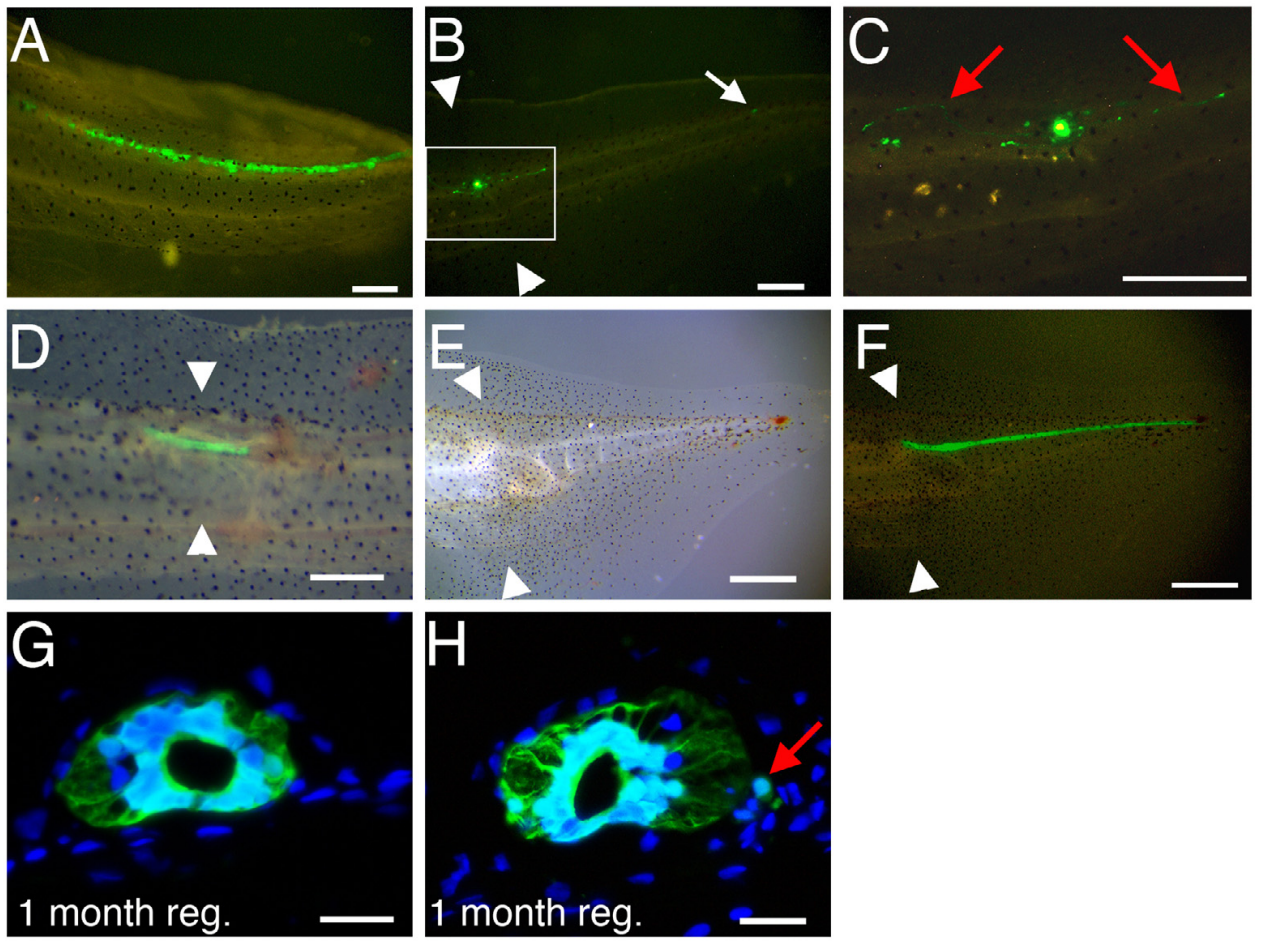
**Tail regeneration after spinal cord labelling or grafting**. (A) Electroporation of GFP plasmid into the spinal cord lumen labels some spinal cord cells. (B) GFP+ cells were observed only within the regenerating spinal cord. (C) Enlarged view of the section in (B), showing neurons with GFP+ve axons. (D-F) Spinal cord grafting in stage 48 tadpole (D) and its regeneration (E, F). GFP was only observed in spinal cord of the regenerate (F). White arrowheads indicate amputation level. Scale bars: 500 μm. (G, H) GFP expression in the regenerating tail from spinal cord-grafted tadpoles. GFP is green and nuclei are stained with DAPI (blue). In this case a single cell with GFP expression is found outside of the regenerating spinal cord (red arrow in H). Scale bars: 20 μm.

In our previous work [3] we examined the fate of labelled spinal cord and showed that no cells left it during regeneration. However these experiments were conducted in such a way that only the ventral part of the cord was labelled and if new neural crest is being formed it is more likely to appear on the dorsal side. To create a more uniform label in the spinal cord we used two new methods: electroporation and grafting of labelled tissue at the tadpole stage. In the first method, a *GFP *plasmid was injected into the tadpole spinal cord lumen and delivered to the lateral or dorsal wall by electroporation (Fig. 1A). 24 hours later, the tadpoles were amputated at the site of GFP expression, and the tail regenerate was examined. In all 29 specimens, the expression of GFP was observed solely within the regenerating spinal cord and no GFP-positive cells were observed outside it (Fig. 1B, C; Table 1).

**Table 1:** Non emigration of cells from the spinal cord

		GFP label of:	
Labelling method	Number of cases	Spinal cord cells	Other
Electroporation	29	29	0
Graft	27	27	2

Since electroporation labels only a small proportion of the cells in the spinal cord it is possible that export of a limited number of cells would not be observed in these experiments. For this reason we have also used spinal cord grafting. Here the donors were *CMV-GFP *transgenics which have previously been shown to express GFP ubiquitously and permanently [3]. This method has the advantage that all the donor cells are labelled, but the disadvantage that contamination from adhering non-neural tissues is impossible to exclude with certainty. To carry out the graft, a piece of spinal cord in non-transgenic tadpoles is replaced with an equivalent piece from a *GFP *transgenic (Fig. 1D). When these tails were amputated at a level such that at least 500 μm GFP spinal cord remained in the stump, the regenerated spinal cord is green along its entire length, showing that it is derived from the donor spinal cord (Fig. 1E,F). In 25 out of 27 cases the labelled cells were entirely confined to the spinal cord even though the tadpoles were allowed a long period of regeneration time and fixed just before metamorphosis (Fig. 1G). Only 2 grafts yielded any GFP-expressing cells outside the spinal cord. One showed some cells in the dorsal fin and the other near the ventral spinal root exit point (Fig. 1H). Because with this protocol it is not possible to guarantee that the grafts are free from contamination by cells outside the spinal cord, we do not regard these two cases as significant. The absence in the great majority of cases of any cells migrating from the spinal cord during regeneration argues against the reformation of a neural crest population.

### Spinal ganglia are not regenerated

**Figure 2:**
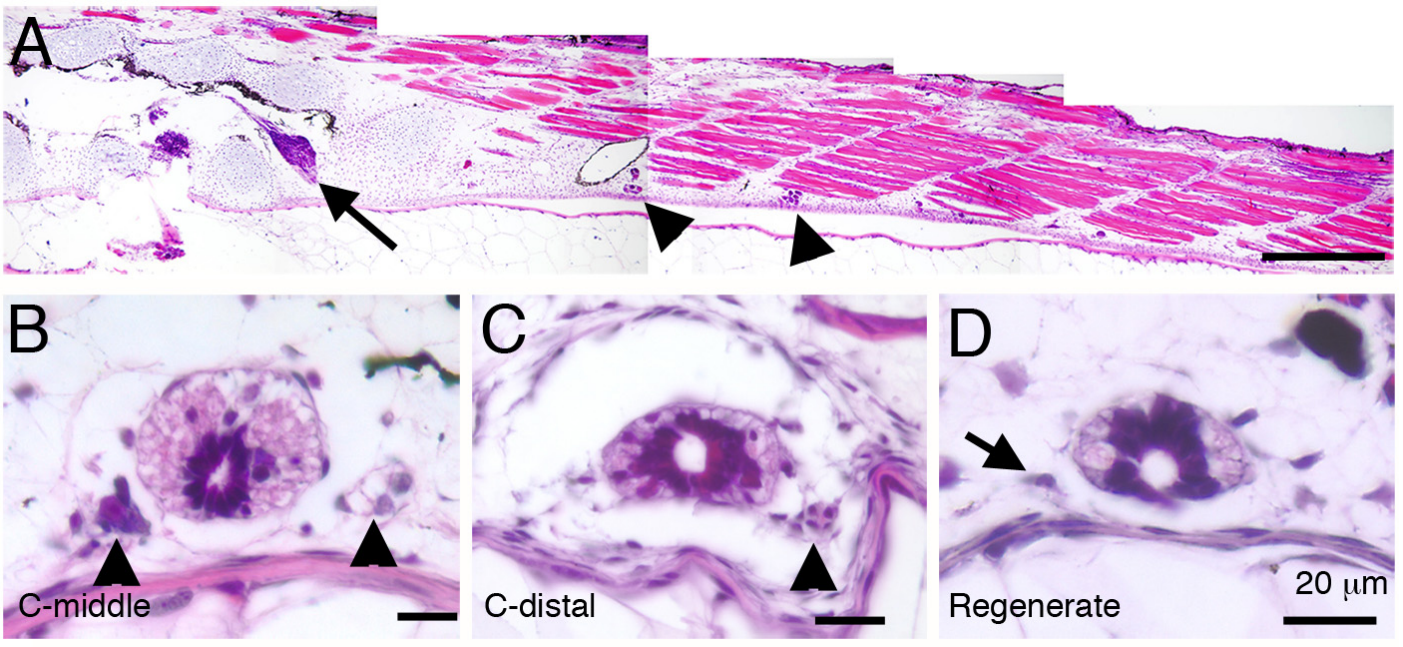
**Morphology of the spinal ganglia viewed by haematoxylin-eosin staining**. (A) A parasagittal section of the dorsal root ganglia in a stage 49 *Xenopus *tadpole is shown. The black arrow indicates ganglion 9. (B-D) Transverse sections of middle (B), distal (C) and regenerated (D) *Xenopus *tails. Arrow heads indicate the spinal ganglia in (B, C) and the arrow in (D) shows a "sporadic ganglion cell" in a regenerate. Scale bars: 500 μm in (A), 20 μm in (B-D).

We also made a study to look for the presence of spinal (= dorsal root) ganglia in the regenerate. As shown in Fig. 2, in stage 49/50 tadpoles, the spinal ganglia in the trunk are large (at least 60 μm long) and are clearly distinguishable. Ganglion 9, the last of the lumbar ganglia, is found at the level where the spinal cord diminishes sharply in size with enlargement of the central canal (arrows in Fig. 2A). At more caudal levels, the spinal cord flattens and the ganglia are much smaller in size, only visible as cell clusters (arrow heads in Fig. 2A). In the middle of the tadpole tail (i.e. 50% postanal distance), a spinal ganglion typically consists of 10–20 cells in a cluster about 20 μm in diameter (Fig. 2B). The presence of these cell clusters continues to the distal part of the tail, where each cluster consists of only 3–5 cells (Fig. 2C). In regenerating tails collected one week after amputation, none contained structures resembling the spinal ganglia of the normal growing tails. When more advanced tail regenerates were examined only 2 out of 24 possessed any cell clusters comparable to those found in control tadpoles (Table 2). In most cases, we only found single cells occupying the position between the spinal cord and the notochord (Fig. 2D). These cells were previously observed by Filoni [2] and referred to as "sporadic ganglion cells". This result shows that the *Xenopus *tadpole does not reconstitute normal spinal ganglia after tail amputation, although occasional extramedullary sensory neurons may be present.

**Table 2:** Presence of tail spinal ganglia in *Xenopus *tadpoles

Specimen	Sections with ganglion	Sections without ganglion	Tails with ganglia	Number of Tails	% tails with ganglia
Control	218	264	6	6	100
1 week regenerate	0	1696	0	15	0
2 week regenerate	23	1296	1	12	8.3
1 month regenerate	54	2592	1	12	8.3

### Neurons and axons are present in the regenerated tail

**Figure 3:**
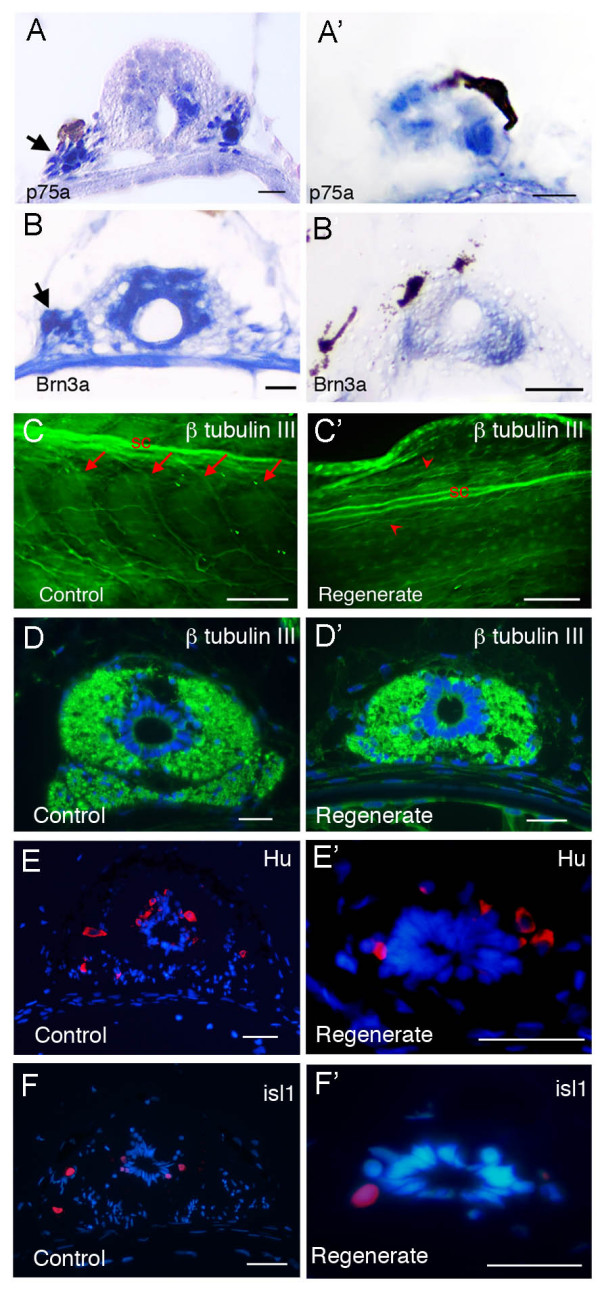
**Expression of neural markers in spinal cord of normal and regenerating tails**. (A-B') *In situ *hybridization detection of mRNA expression of *p75a *(A, A') and *Brn3a *(B, B') on transverse sections. (A-B) Control tails. (A'-B') Tail regenerates. Arrows in (A, B) indicate ganglia and the arrow in (A') indicates a sporadic ganglion cell. (C-D') β III tubulin expression (green) in whole mount tadpole tails (C, C') and on cross sections (D, D'). (C', D') are one-month regenerates. (E-F') Expression of Hu (E, E') and islet 1 (F, F') are detected by antibody staining on transverse sections. (E, F) un-operated control, (E', F') 2 week old tail regenerates. Scale bars: 250 μm in (E, E'), 20 μm in the rest.

Despite the absence of typical sensory ganglia in the regenerate it is possible that sensory neurons are regenerated and remain within the spinal cord. To investigate this we examined the expression of genes encoding a neurotrophin receptor p75, and Brn3a, an important transcriptional regulator in sensory neurons [32-34] by *in situ *hybridization. Both are expressed in the normal dorsal root ganglia and in a lateral position in the regenerating spinal cord (Fig. 3A–B').

The tails were also immunostained for the neuron-specific beta III tubulin in order to reveal the overall arrangement of the fibre tracts and the neuronal cell bodies. In lateral view (Fig. 3C–D') it can be seen that the segmental pattern of axons seen in the original tail is not regenerated, and the axons innervating the regenerate are coming from more anterior levels. Transverse sections (Fig. 3D, D') show the presence of fairly similar fibre tracts in the original and regenerated spinal cord. Expression of Hu protein in the spinal cord of normal growing and regenerating tadpole tails confirms the presence of neurons (Fig. 3E, E')[35]. Some of these are in a dorsal location and so are probably sensory neurons. Others are in a lateral or ventral location and so are probably motor neurons. Some of these express the transcription factor islet1 (Fig. 3F, F') [36].

**Figure 4:**
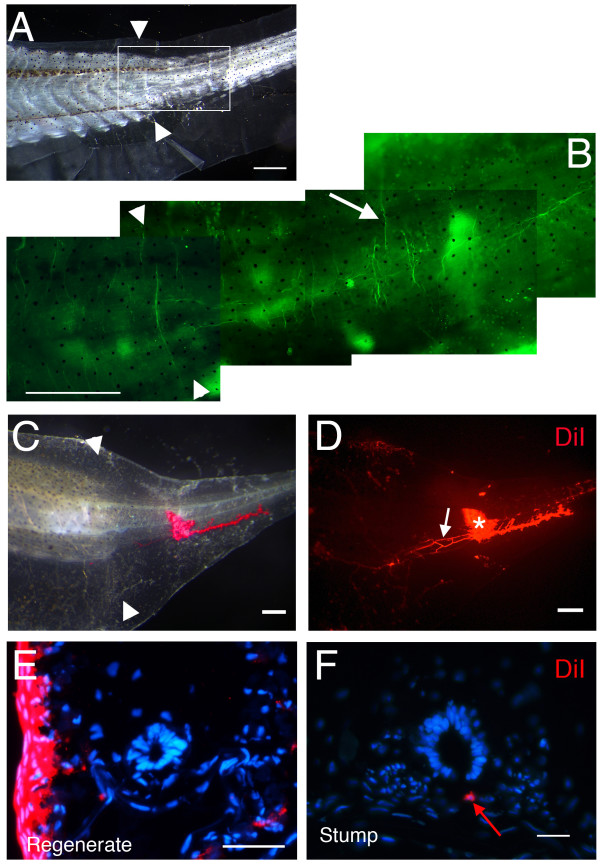
**Peripheral nerve fibres in tail regenerates**. (A) 1 month old tail regenerate. (B) Neurofilament 200 staining, enlarged view from the section in (A). Arrow indicates a neurofilament 200 positive nerve fibre. (C) Bright field image of a one week old tail regenerate, after DiI injection. (D) Red fluorescent image of (C), * marks the injection site of DiI, white arrow indicates a DiI-labelled nerve fibre. (E, F) The DiI signal is not found in neurons of the regenerating spinal cord (E) but is found in neurons of the proximal stump (red arrow in F). Arrow heads in (A-C) mark the amputation levels. Scale bars: 500 μm in (A-B); 250 μm in (C, D) and 20 μm in (E, F).

We noticed that a light touch to a regenerated tail provoked an escape response by the tadpole, suggesting the presence of sensory innervation in the regenerate. To further investigate this, we performed whole mount immunostaining on tail regenerates with an antibody recognizing 200 kda neurofilament (Fig. 4A–C). This shows that peripheral axons exist in the regenerated part of the tail (Fig. 4A,B). Retrograde labelling of nerve fibres was carried out by DiI injection just underneath the skin (Fig. 4C,D) and indicates that the regenerating tail is innervated with sensory fibres. As was apparent with the β III tubulin staining, these axons are mostly derived from the spinal cord more anterior to the amputation level, because the DiI can be found in cell bodies of the anterior spinal cord but is lacking in those of the regenerate (n = 7; Fig. 4E, F).

These results indicate that there is abundant sensory innervation in the regenerated tail, despite the great reduction of sensory ganglia. There are neurons, probably both motor and sensory, present in the regenerated part of the spinal cord but, at least for the stages examined, the axons of the regenerate originate from neurons in the stump.

### Melanophores originate from committed precursors

**Figure 5:**
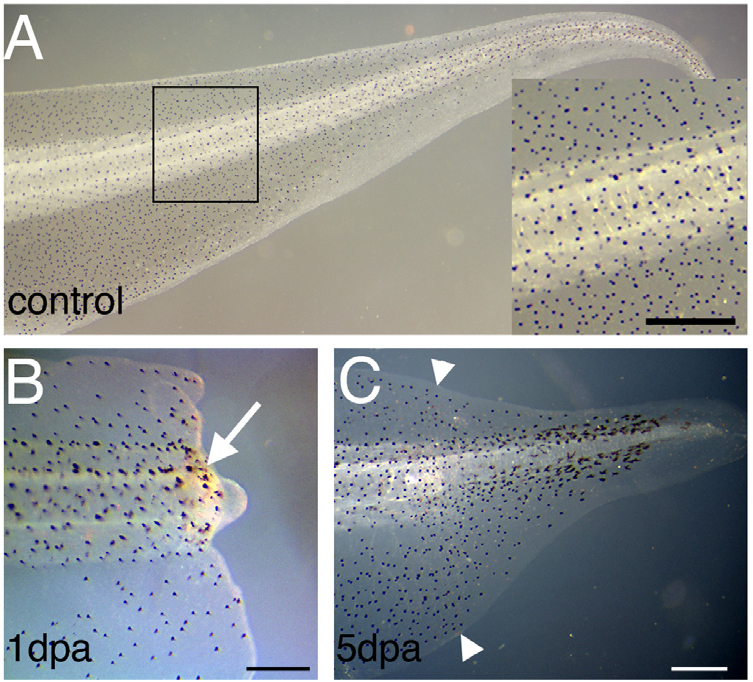
**Regeneration of pigmentation in Xenopus tadpoles**. (A) Pigment pattern of a stage 49 tadpole. The inset is an enlarged view of the amputation level. (B) A regenerating tail 1 day post amputation (dpa). The white arrow indicates the melanophores near the surface. (C) A regenerating tail 5 dpa.

A group of neural crest-derived cells whose presence is obvious in the tail regenerates is the pigment cells. This discussion will concern only the melanophores and not the xanthophores and iridophores, which are less abundant in the tail. The melanophores in a 7-day regenerate (Fig. 5C) are very numerous, similar in density to that of the normal growing tail (Fig. 5A). The cells are seen in the regenerating bud as early as one day after amputation (white arrow, Fig. 5B). We reasoned that there might be three possible cell sources for the melanophores in the tail regenerate.

Firstly, as in early development, they may be derived from the spinal cord. Secondly, they might arise from some sort of pluripotent stem cell located in the dermis. Thirdly, as shown in the zebrafish, the regenerated melanophores might arise from pre-existing melanophore precursor cells present in the tail stump[30]. Experiments were carried out to test each of these three possibilities, and only the last is supported.

#### Melanophores arise from embryonic neural crest but not from tadpole spinal cord

**Figure 6:**
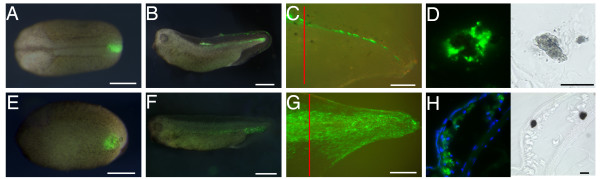
**Tail regeneration after embryonic grafting of neural crest or of posterior ventral epidermis+mesenchyme**. (A-C) A piece of *GFP *transgenic posterior neural crest was grafted to the same positions in wild type hosts. The labelled embryos were grown to tadpoles (B), amputated at stage 48 and allowed to regenerate for 7 days (C). Red lines indicate the amputation level. (D) A single melanophore in a regenerated tail from such an experiment, with contracted pigment and abundant GFP in the cytoplasm. (E-H) Similar experiment with graft of posterior ventral epidermis+mesenchyme (PVEM). In (H) is shown a section of the regenerate with two melanocytes, neither of which is labelled. White scale bars 500 μm; black scale bars, 20 μm.

When grafts are made of the neural fold region of neurula stage embryos (Fig. 6A) many melanophores become labelled, as well as fin mesenchyme and the spinal cord itself. This is shown in Fig. 6A–D and in the first line of Table 3. Grafts were taken from *CMV-GFP *transgenic donor and were orthotopic, replacing a similar piece of tissue in the host embryo at stage 15–17. In the second line of Table 3 are shown a different series of grafts from the centre of the neural plate, similar to those previously described in [3]. These do not label the melanophores because the graft populates just the ventral half of the neural tube and not the neural crest. This confirms, as expected, that the melanophores do arise from the neural crest in embryonic development, and that they do not arise from the ventral neural tube. It also confirms that labelled melanophores can readily be observed in the regenerates despite the presence of deep pigmentation (Fig. 6D). During fixation the pigment tends to contract towards the nuclei leaving the peripheral region of the cell unobscured so the GFP can be visualised.

**Table 3:** Origin of melanophores

Graft	Number of cases	Spinal cord	Fin	Muscle	Melanophores
Embryo neural crest	36	12	33	0	29
Embryo central neural plate	28	28	0	0	0
Embryo posterior ventral ectomesenchyme	28	0	13	5	0
Tadpole skin	22	0	0	13	17

In contrast to the grafts of embryonic neural crest, the experiments presented above (Fig. 1 and Table 1), of labelling by electroporation or grafting of the tadpole spinal cord followed by amputation, did not normally result in the formation of labelled melanophores in the regenerate. In fact only one case out of the 27 spinal cord grafts did so and since this type of experiment cannot guarantee the absence of contamination by surrounding tissues this individual case is not considered significant.

#### Melanophores are regenerated from tadpole skin but not from dermal fibroblasts

**Figure 7:**
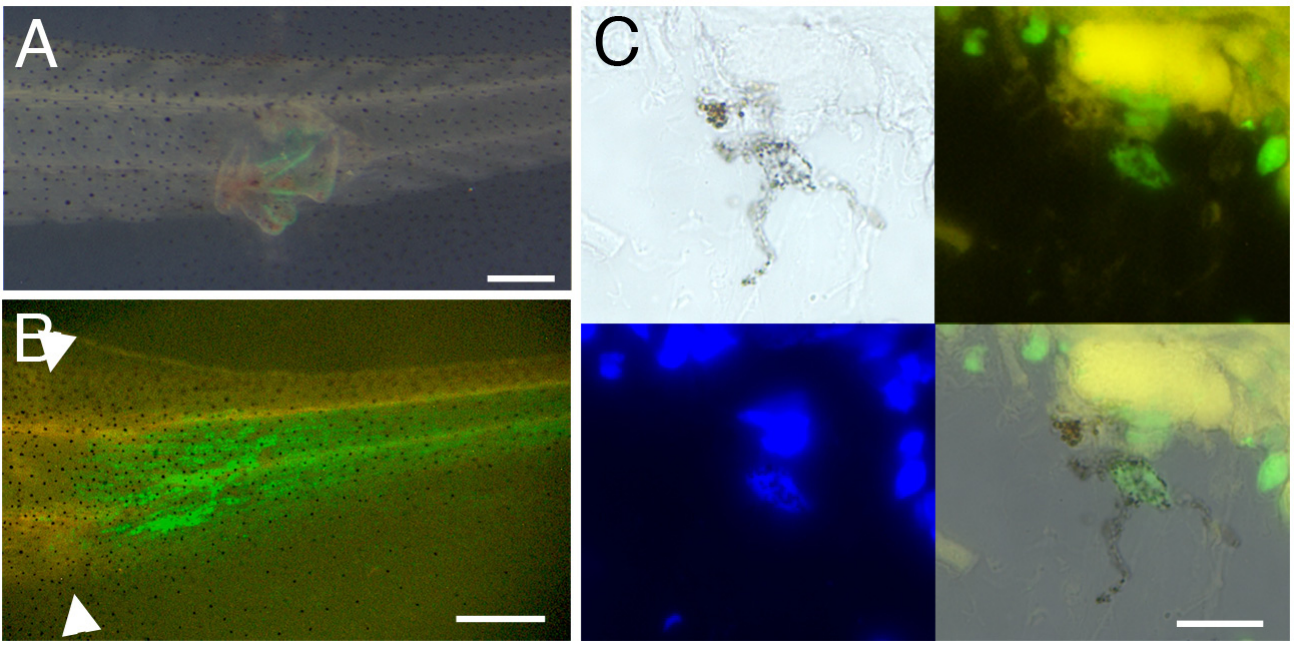
**Regeneration of melanophores after skin grafting in tadpoles**. (A) A piece of GFP-labelled skin was grafted to the lateral region of the middle trunk of a non-GFP tadpole host, which was then amputated 3 days after. (B) A 7 day tail regenerate from a skin grafted tadpole, white arrowheads indicate the amputation level. (C) Detection of GFP in a melanophore in the regenerate. 4 views are shown: top left transmitted light; top right GFP fluorescence; bottom left DAPI fluorescence (DNA); bottom right transmitted light and fluorescence. Scale bars: 500 μm in A, B, 10 μm in C.

If melanophores do not come from the spinal cord, then where do they come from? The next set of experiments involved skin grafts from *CMV-GFP *transgenic donor tadpoles to wild type hosts. After healing for 2 days, the tails were amputated through the graft (Fig. 7A) and the GFP expression was followed for 14 days (Fig. 7B). Following this type of graft GFP-positive melanophores are regularly found (Fig. 7C, Table 3). It follows that some cell type present in the skin is the precursor of the melanophores, but skin contains a whole variety of cell types including the epidermis, the dermal fibroblasts, the blood vessels, the pre-existing melanophores, and perhaps also mesenchymal stem cells of some sort. So according to this experiment any of these might be the precursors.

To eliminate most of these possibilities, we performed early embryonic grafts of the posterior ventral ectomesenchyme (PVEM) of stage 15–17 *CMV-GFP *transgenics to wild type hosts (Fig. 6E). With such grafts a broad lateral region of the developing tail is labelled and the epidermis and dermis are both labelled, but not the melanophores (Fig. 6F). This type of graft sometimes also labelled a few myotomes, indicating contamination with muscle precursor cells. When amputated through the grafted area, the tail regenerate contains a great number of GFP-positive cells. As in the original tails, the epidermis and mesenchyme cells are labelled (Fig. 6G–H), but not the melanophores. Thus this experiment excludes an origin from epidermis or from any cell type derived from embryonic dermal mesenchyme, including any possible pluripotent stem cells, in the regeneration of the melanophores.

#### Melanophores are regenerated from pre-existing neural crest-derived precursors

The last possibility for the origin of the melanophores in the regenerate is the pre-existing melanophores or melanophore precursors near the amputation site. This seemed likely because the tadpole skin grafts do contain labelled melanophores, while the tails generated from embryonic grafts of PVEM do not. However, we could not be sure that there was not also some other difference in labelled cell composition between these two types of experiment.

**Figure 8:**
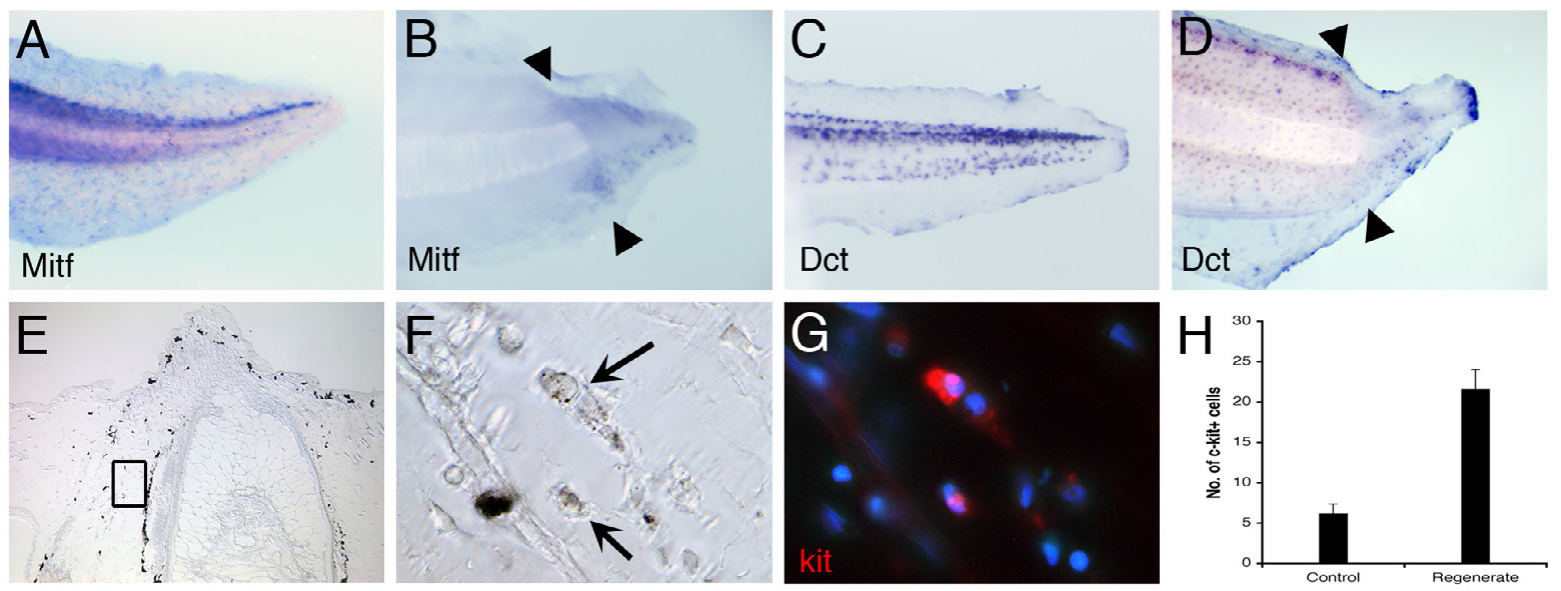
**Detection of *mitf*, *dct *and kit expression**. (A-D) Expression of *mitf *(A, B) and *dct *(C, D) transcripts in normal tadpole (A, C), and 3d tail regenerates (B, D) detected by *in situ *hybridization. Positive cells are present in the blastema region of the regeneration bud. Black arrows indicate amputation level. (E-H) Detection of kit in tail regenerates. Enlarged view of the selected area in (E) is shown in (F). The kit antibody staining is shown in red, and counterstained with DAPI (G). (H) Quantification of kit^+ ^cells in the stump and regenerating tails, n = 6.

To look for melanophore precursor cells in the tadpole tail we examined normal and regenerating tails by *in situ *hybridization for the expression of three melanoblast markers. They are the early melanoblast markers *mitf *(microphthalmia-associated transcription factor) [37-39] and *kit *[40,41], and the late melanoblast marker *dct *(dopachrome tautomerase) [22]. Cells expressing all these markers were found both in the normal and the regenerating tadpole tails (Fig. 8).

**Figure 9:**
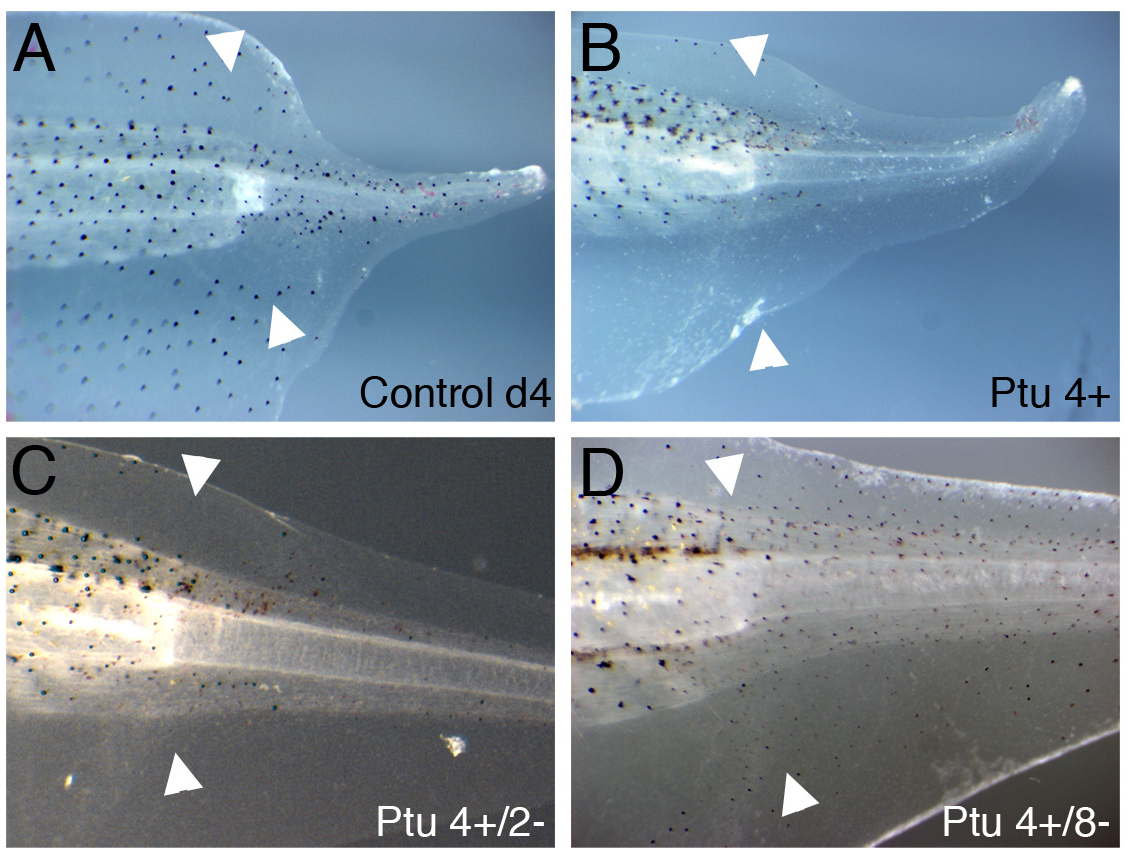
**Tail regeneration in Phenylthiourea (PTU) treated tadpoles**. (A) Untreated 4 day tail regenerate. (B) Tail regenerate of a tadpole treated with PTU for 4 days, starting immediately after tail amputation. (C) same tadpole as in (B), 2 days after PTU withdrawal. (D) same tadpole as in (B), 8 days after PTU withdrawal. White arrowheads mark the amputation level.

To distinguish whether the regenerated melanophores come from pre-existing pigmented melanophores or from un-pigmented melanophore precursors, we used the tyrosinase inhibitor phenylthiourea (PTU) to block melanin synthesis [30]. The tails were allowed to regenerate in the presence of 0.1–0.2 mM PTU, which blocks the appearance of any melanophores in the regenerate. When the PTU is removed the tadpoles acquire pigmented cells in their regenerates over about a week (Fig. 9). This process is slower than in the zebrafish but the distribution of the cells is random across the regenerate, not appearing first at the proximal end as would be expected if melanophores were simply migrating from the stump region. These result suggest that the major source of the regenerating melanophore is the unpigmented melanophore precursor cells in the tail region.

**Figure 10:**
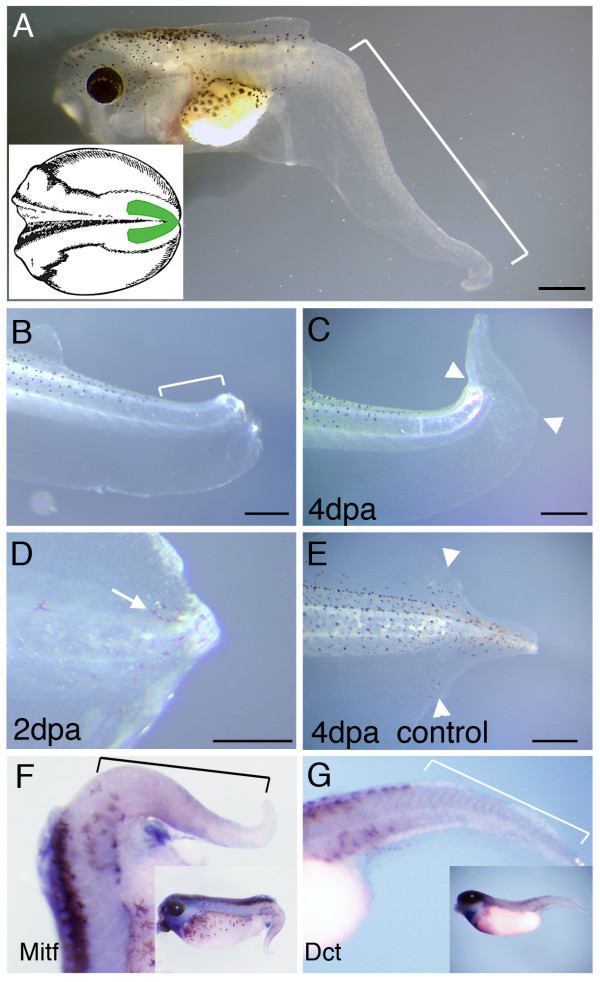
**Tail regeneration in normal and neural crest-extirpated tadpoles**. (A) A tadpole developed from a neural fold-extirpated embryo. The white bracket indicates the tail region with its pigment cells and dorsal fin depleted. The inset is a sketch of a stage 15/16 embryo, the green area marking the posterior neural fold removed. (B) A tadpole immediately after amputation. The white bracket indicates the region free from melanophores. (C) The same tadpole as in (B), 4 days after tail amputation, arrowheads indicate amputation level. (D) A tadpole with reduced number of melanophores close to the tail amputation level, 2 days after amputation. Arrowheads indicate amputation level. (E) A control tadpole, 4 days after amputation. White arrowheads indicate amputation level. (F, G) *In situ *hybridization of *mitf *(F) and *dct *(G) in neural fold extirpated tadpoles. Brackets mark the melanophore-free region of the tail. Scale bars, 500 μm.

Previous work in our lab has shown that removal of the posterior neural fold of neurula stage embryos (inset in Fig. 10A) disrupts the development of the dorsal fin and creates a melanophore-free region in the tadpole tail [42]. Although it is not easy to make these tadpoles, a series of 16 was successfully prepared with complete absence of melanophores from the tail (Fig. 10A, white bracket). When these tadpoles were amputated through the melanophore-free region, at a distance more than about 500 μm away from the proximal visible melanophore (Fig. 10B), no melanophores appeared in the regenerated tail (Fig. 10C). In contrast, melanophore regeneration was observed in all control tadpoles 3 days after amputation (Fig. 10E). *In situ *hybridization of *mitf *and *dct *in these neural fold extirpated tadpoles showed a normal pattern in the anterior but an absence of positive cells in the region of the tail affected by the neural fold extirpation (fig. 10F,G). This experiment is the complement of the skin graft experiments, it shows that in the absence of neural-crest derived cells then no melanoblasts regenerate. Our conclusion therefore is that in embryonic development the melanophores arise from the neural crest, while in regeneration they arise from pre-existing melanophore precursors.

## Discussion

Using two methods of labelling we show that there is no emigration of any cell population from the spinal cord during regeneration, which indicates that there is no recapitulation of neural crest formation as it occurs in the embryo. In embryonic development the neural crest is induced through a reciprocal interaction between the epidermis and the neural plate, both parts of a common ectodermal cell sheet during the neurula stages [15-19]. The neural crest cells exist for a period in the folds of the neural plate, and then after closure in the dorsal neural tube. Following this they migrate out of the central nervous system to reach their final positions. The anatomical situation of regeneration does differ in several respects from that of early development. In particular, in regeneration there is no contact between the epidermis and the transected end of the spinal cord, which closes to form the neural ampulla, and so there is no opportunity for a neural crest inductive event to take place for a second time. In view of this it is perhaps not surprising that the process of regeneration of neural crest derivatives does not recapitulate their original embryonic development.

In urodele amphibians the tail will often regenerate in adult as well as larval life. Instead of a notochord the larval and adult urodeles have segmented vertebrae composed of cartilage or bone. However, the actual regeneration bud looks relatively similar to *Xenopus*, with a distinct neural ampulla, cartilaginous rod in the vertebral region, and loose blastema-like cells surrounding them [43]. In urodele tail regeneration the spinal ganglia are reformed [9]. Although there is the same anatomical segregation of tissue types in the urodele, it has been shown that cells of the spinal cord ventricular layer can be labelled by electroporation and their progeny may later appear outside the spinal cord having undergone metaplasia to cell types other than neurons or glia [8,44]. This implies that in urodeles at least some cells of the spinal cord can migrate away and can de-differentiate, and this process may underlie the regeneration of the spinal ganglia. De-differentiation is of course also found in some other regeneration situations in urodeles, including the lens and the limb [45,46].

We show that the *Xenopus *tail regenerates do not contain well formed spinal ganglia, although there may be a few extramedullary neurons. These extramedullary cells presumably do originate from the spinal cord but are few in number and found only in a minority of individuals. In previous work by Filoni's group it was shown that *Xenopus *spinal ganglia can regenerate, but their study examined only the large brachial and lumber level ganglia of the trunk [47]. Interestingly the ganglia did not regenerate if they were simply ablated, but they did regenerate if a segment of the spinal cord was also ablated at the same level, with the new ganglion cells coming from the spinal cord. Similar behaviour is shown in urodeles, where spinal ganglia are regenerated following transection of the tail but not following simple removal [9]. The situation we find in the *Xenopus *tail is obviously different both from the urodele and from the trunk ganglia in *Xenopus *itself.

There are neurons in the regenerated spinal cord, visualised by Hu and β-III tubulin staining, and abundant innervation of the regenerated tail, visualised by neurofilament or β-III tubulin staining. The dorsally located neurons of the regenerate may include Rohon Beard neurons. These are large sensory neurons within the spinal cord, not generally considered to be of neural crest origin, which are formed very early in development and whose numbers gradually decline thereafter [48,49]. Apart from dorsal position, more definite evidence for sensory innervation is provided by the presence of cells within the regenerated spinal cord which express genes for the neurotrophin receptor p75 and the transcriptional regulator Brn3a [32,50], and by the retrograde labelling result with DiI injected into the skin (Figs. 3, 4). In the regenerates the pattern of spinal nerves, visualised both by immunostaining and by DiI tracing, does not show the regular segmental arrangement of the original tail. The results suggest that, despite the presence of neurons in the regenerated spinal cord, much of the innervation for the regenerate comes from neurons at more anterior levels. This is similar to the motor innervation of the normal tail, and to the arrangement of both motor and sensory innervation in some anuran species other than *Xenopus *[51].

Overall, the regeneration of a spinal cord containing sensory neurons, and the sensory innervation of the skin and muscle of the new tail, indicate that an adequate sensory system is being regenerated even though it is not an exact copy of the original and does not arise via the re-formation of an obvious population of neural crest cells.

The lizard is the "highest" vertebrate able to regenerate a tail [52]. Here the spinal cord does regenerate, but consists only of descending fibre tracts, without neurons. No spinal ganglia are regenerated and the sensory innervation to the regenerate arises from the last three remaining ganglia of the stump [53]. So the *Xenopus *tadpole lies in between the lizard and the urodele in terms of completeness of the spinal cord regenerated.

### Melanophore regeneration

Our results reported here show that the melanophores in the regenerating tail come from the melanoblasts, or other neural crest derived precursors in the stump, and not from the spinal cord or from non-neural crest-derived cell sources. The evidence for this is straightforward. Firstly, we show that cells near the amputation level express *kit*, *mitf *and *dct*, markers of various stages of melanoblast [30,40,41](Fig. 8). Secondly, we show that tails regenerated from GFP-skin-labelled tadpoles contain GFP-expressing melanophores (Fig. 7). Thirdly, grafting of the posterior lateral ectomesenchyme of neurula stage embryos does not give rise to GFP-labelled melanophores in the regenerating tails, showing that there is no contribution of the epidermis or the dermal cells to melanophore regeneration (Fig. 6). Finally, we created melanophore-free tails by removal of the third of the posterior neural fold in early neurulae [42], and we show that if there is no melanophore near the stump, then there is no melanophore regeneration in the tail (Fig. 10). These results are comparable to those previously obtained in the zebrafish, also showing that melanophores regenerate from unpigmented precursors [30,31,40]. It is also consistent with what is seen in wound healing of human skin, where the re-colonization of the epidermis involves the migration of melanocytes from the immediately adjacent epidermis [54].

## Conclusion

This work indicates that the regenerated tadpole tail is not a faithful copy of the original, despite its functional capacity that enables the tadpoles to swim. One way in which it differs is in a lack of neural crest derivitives, for which regeneration is confined to the melanophores. Although some sensory neurons are regenerated within the spinal cord, there is no epithelial-mesenchymal transition of dorsal neural cells, no migration of cells out of the spinal cord, no formation of new spinal ganglia, and no formation of fin mesenchyme or melanophores from the spinal cord.

The regeneration of melanophores from pre-existing melanophore precursors provides a further example of tissue-specific regeneration without metaplasia, similar to the previously described regeneration of spinal cord, notochord and muscle. We conclude that the regeneration of the tadpole tail does not proceed by a wholesale reversion to an embryonic state, nor through a mobilisation of pluripotential stem cells present in the tail. Instead tadpole tail regeneration seems to occur through a modification of the normal ongoing processes of tail growth. In regeneration the growth rates are somewhat increased for each of the cell populations such that they form a new tapered appendage rather than simply expanding with preservation of overall shape as they would in the normal growing tail.

## Methods

### Embryos and tadpoles

Wild type *Xenopus *laevis embryos were obtained by *in vitro *fertilization. *GFP *transgenic embryos were made by transgenesis with a *pCNA3-CMV-nucGFP *construct linearized with *Sma*I. The transgenics were made by sperm nuclear injection according to the method of [55], with modifications as in [56]. Embryos were staged according to the Nieuwkoop and Faber (NF) tables [29]. Embryos were dejellied with 2% cysteine (Sigma) pH 7.8, and then cultured in 0.1 × NAM. Transgenics were identified by virtue of GFP expression. From stage 46, they were transferred to recirculating aquarium and fed on tadpole diet (Blades Biological, Redbridge, UK) twice a week. To inhibit melanin synthesis 0.1–0.2 mM phenylthiourea (PTU) was used.

### Grafting, neural fold extirpation and tail amputation

Grafts on early stage embryos were performed as described previously [3]. For tadpole spinal cord grafting, stage 48+ wild type and *GFP *transgenic tadpoles were anaesthetized in 0.02% MS222, and were kept in the anaesthetic solution during the operation. A small piece of spinal cord in the middle tail was removed by two small incisions with a sharp microsurgery knife, cutting through the lateral muscle. The donor *GFP*+ spinal cord piece was inserted into the wild type receipt and covered with a piece of cover slip for 15 minutes before recovery. The skin grafts in tadpoles were performed similarly.

To obtain melanophore-free tadpoles, neurula stage embryos were transferred into full strength NAM solution. The posterior neural fold was removed with a hair knife and the embryos were cultured in NAM/2 at 18°C.

For tail amputation, the tadpoles were anaesthetized in 0.02% MS222 and the distal 50% of the tail was removed with a pair of iridectomy scissors (Vannas straight small, John Weiss). The tadpoles were allowed to heal in tap water with aeration (1 hour) and subsequently transferred to the aquarium.

### Electroporation

The tadpoles were anaesthetized in 0.02% MS222 solution. A small amount (<1 μl) of *pCNA3nucGFP *plasmid (1 μg/μl, in water) was injected into the spinal cord lumen via the brain. Square pulses of 50 v, 10 msec were applied three times with a 100 msec intervals, using an Electro Square Porator (ECM 830, BTX) and homemade electrodes. The tadpoles were then allowed to recover and transferred to the aquarium.

### *In situ *hybridization

Whole mount *in situ *hybridization was performed according to the standard protocol [57]. On section *in situ *hybridization was performed as described by [58]. Probes were prepared with *SP6 *or *T7 *RNA polymerase from linearised constructs. These are *p75a *(*Apa*I, *SP6*), *Brn3a *(*Ap*aI, *SP6*), *dct *(*Sac*I, *T7*) and *mitf *(*Xho*I, SP6).

The constructs to make *p75a, Brn3a *and *dct *probes were obtained by cloning from cDNA of stage 33 embryos. PCR fragments were ligated into pGEMT vector (Promega) and sequenced to identify the orientation of insertion. Primers used were: *Xenopus Brn3a *(**AF196575**), 5'-tatattcgccagtctggatg-3', 5'-tcgggtttgttgagtttttc-3'; *Xenopus dct *(**AB108531**), 5'-tctgtccgggacacattgct-3', 5'-ttcctgaaaaaaggaggatt-3'; *Xenopus p75a *(**AF172339**): 5'-aagcagaacaagcagggaggtaac-3', 5'-atgtgggtggaagagaactacggt-3'.

### Morphology and immunohistochemistry

For morphological studies, tadpoles were fixed in Zamboni's fixative (40 mM NaH_2_PO_4_, 120 mM Na_2_HPO_4_, 2% PFA, 0.1% saturated picric acid) overnight at 4°C, dehydrated and embedded in paraffin. 7 μm sections were prepared, mounted on slides and stained with haematoxylin and eosin.

Immunohistochemistry was performed on paraffin sections prepared as above. Sections were permeabilised in PBS containing 1% Triton X-100 and blocked with 2% Boehringer Blocking Reagent (Roche). Primary antibodies used were: neurofilament 200 (Sigma N4142, 1:80), β-tubulin III (Sigma T2200, 1:100), Hu (Molecular Probes A21271, 1: 250), c-kit (Santa Cruz SC168, 1:100) and GFP (Abcam Ab290, 1:500). The islet 1 antibody (39.4D5) developed by TM Jessell was obtained from the Developmental Studies Hybridoma Bank, University of Iowa. Secondary antibodies (VectorLabs) were used at a dilution of 1:200. The slides were either counter-stained in Mayor's haematoxylin (Fluka) and mounted in Depex (BDH), or counter-stained with DAPI and mounted with Gel mounting medium (Biomedia) before observation under the microscope.

Whole mount immunohistochemistry was carried out as described previously [10]. The second antibody used was FITC conjugated anti-rabbit IgG (Vector Labs, 1:250). After staining of the nuclei with DAPI, the whole mount preparation was observed under a Leica stereomicroscope or Leica DMRB microscope.

### DiI retrograde labelling

DiI is a lipophilic dye widely used for tracing pathways in the nervous system [59]. 10 nl of DiI solution (2 mg/ml, in DMSO) was injected into the muscle or underneath the skin of the regenerating or normal growing tadpole tails. Labelling of the nerve fibres was identified by observing under Leica Fluo III dissecting microscope, with or without injection of FITC into the heart of the tadpoles to distinguish the nerve fibres from blood vessels.

### Photography and microscopy

GFP was observed in live tadpoles after anaesthesia in 0.02% MS222, using a Leica Fluo III fluorescent dissecting microscope with a GFP2 filter set. DiI labelling was observed with a TRITC set. Stained sections were visualized with a Leica DMRB microscope. Images were captured using a SPOT RT camera (Diagnostic instruments) and processed with Photoshop software (Adobe).

## Authors' contributions

GL and YC performed the experiments. JMWS conceived the work and supervised it. The paper was written by GL and JMWS and the content is agreed by all the authors.
